# Supplementary material for: Dispersal and fire limit Arctic shrub expansion
Source: Nat Commun. 2022 Jul 4;13:3843. doi: 10.1038/s41467-022-31597-6 (PMC9253140; doi:10.1038/s41467-022-31597-6)
Supplement: Supplementary file 1 — Supplementary Information File [file 41467_2022_31597_MOESM1_ESM.pdf]

Supplementary Information for "Dispersal and fire limit Arctic shrub expansion"

Yanlan Liu[1,2]*, William J. Riley[3], Trevor F. Keenan[3,4], Zelalem A. Mekonnen[3], Jennifer A. Holm[3], Qing Zhu[3], Margaret S. Torn[3]

[1]School of Earth Sciences, The Ohio State University, Columbus, OH, USA

[2]School of Environment and Natural Resources, The Ohio State University, Columbus, OH, USA

[3]Climate and Ecosystem Sciences Division, Lawrence Berkeley National Laboratory, Berkeley, CA, USA

[4]Department of Environmental Science Policy and Management, University of California, Berkeley, CA, USA

*Email: liu.9367@osu.edu

Table of Contents

- Supplementary Table 1
- Supplementary Figures 1-10

Supplementary Table 1 | The Full set of candidate climate variables. Details of each variable were described in Wang et al. (2016).

Variable name and unit
- Mean annual temperature (°C)
- Mean warmest month temperature (°C)
- Mean coldest month temperature (°C)
- Temperature difference between MWMT and MCMT, or continentality (°C)
- Mean annual precipitation (mm),
- May to September precipitation (mm),
- Annual heat-moisture index (MAT+10)/(MAP/1000))
- Summer heat-moisture index ((MWMT)/(MSP/1000))
- Degree-days below 0°C, chilling degree-days
- Degree-days above 5°C, growing degree-days
- Degree-days below 18°C, heating degree-days
- Degree-days above 18°C, cooling degree-days
- Number of frost-free days
- Frost-free period
- The day of the year on which FFP begins
- The day of the year on which FFP ends
- Precipitation as snow (mm). For individual years, it covers the period between August in the previous year and July in the current year.
- Extreme minimum temperature over 30 years
- Extreme maximum temperature over 30 years
- Hargreaves reference evaporation (mm)
- Hargreaves climatic moisture deficit (mm)
- Mean annual solar radiation (MJ m^{-2} d^{-1})
- Mean annual relative humidity (%)

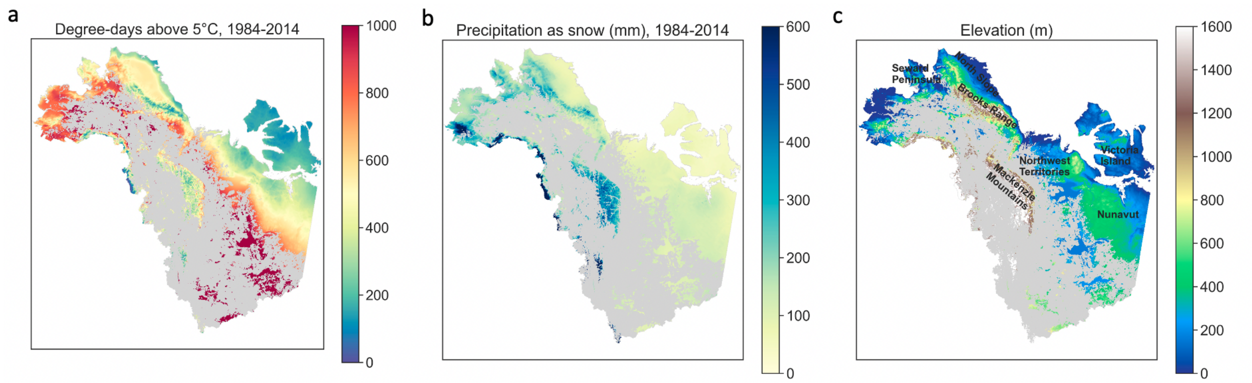

Supplementary Figure 1 | Spatial patterns of the dominant climate and topographic conditions on environmental suitability. Average (a) degree-days above 5 °C and (b) precipitation as snow during 1984-2014. (c) Elevation across the domain, illustrated using a 4 km resolution.

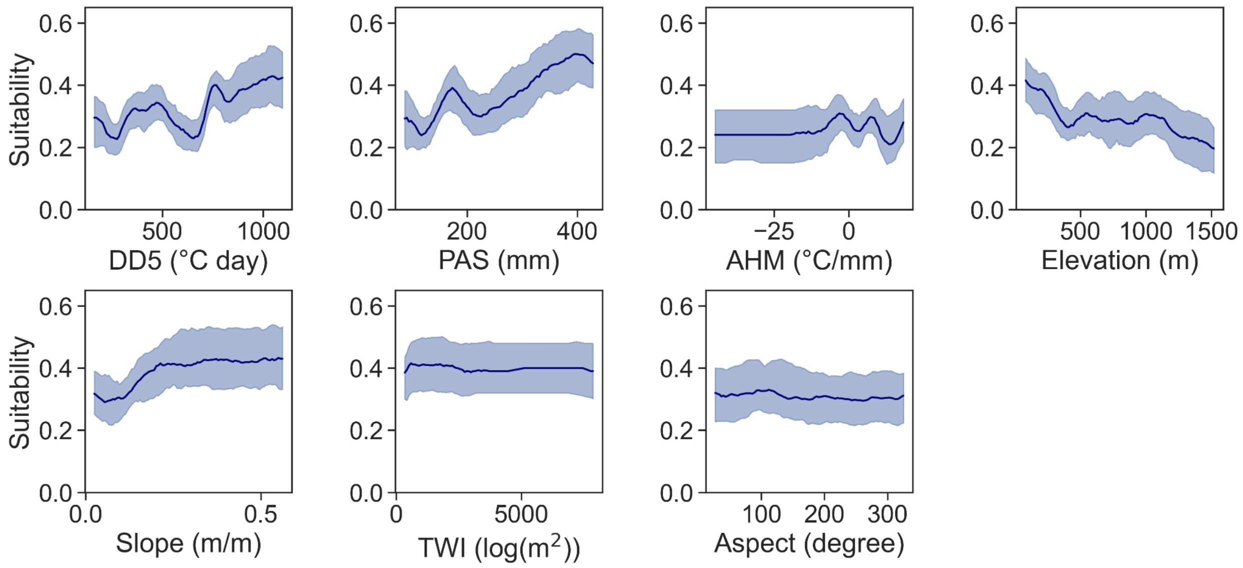

Supplementary Figure 2 | Response curve of environmental suitability to climate and topographic conditions. The conditions include annual degree-days above 5 °C (DD5), annual precipitation as snow (PAS), annual heat-moisture index (AHM), elevation, slope, topographic wetness index (TWI), and aspect. The line and shaded band in each panel show the median and 5%-95% uncertainty range uncertainty across 100 random forest runs.

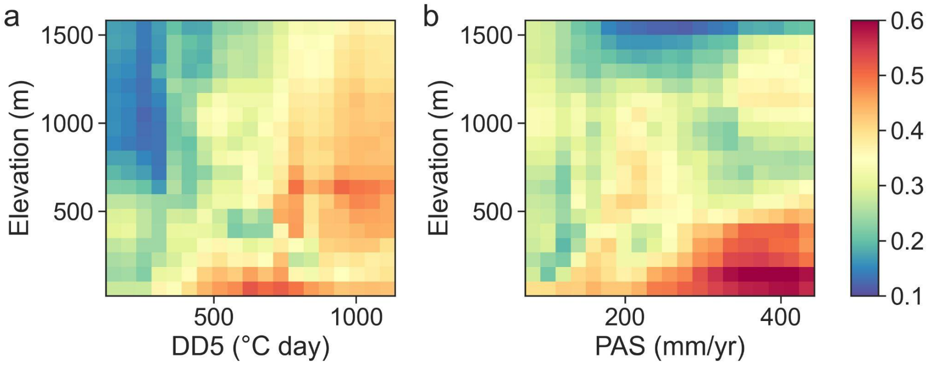

Supplementary Figure 3 | Response surface of environmental suitability to annual degree-days above 5 °C (DD5), annual precipitation as snow (PAS), and elevation.

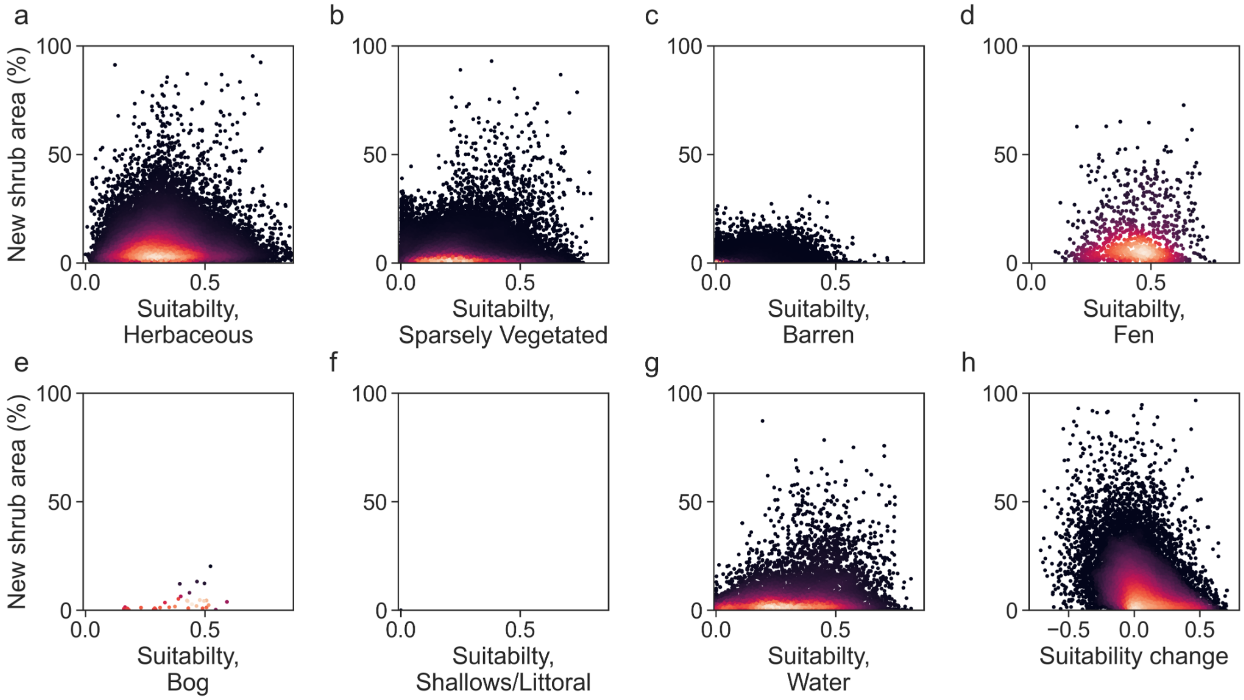

Supplementary Figure 4 | Relationships of new shrub area in 2014 with environmental suitability in different initial land cover types in 1984, and with environmental suitability change between 1984-2014.

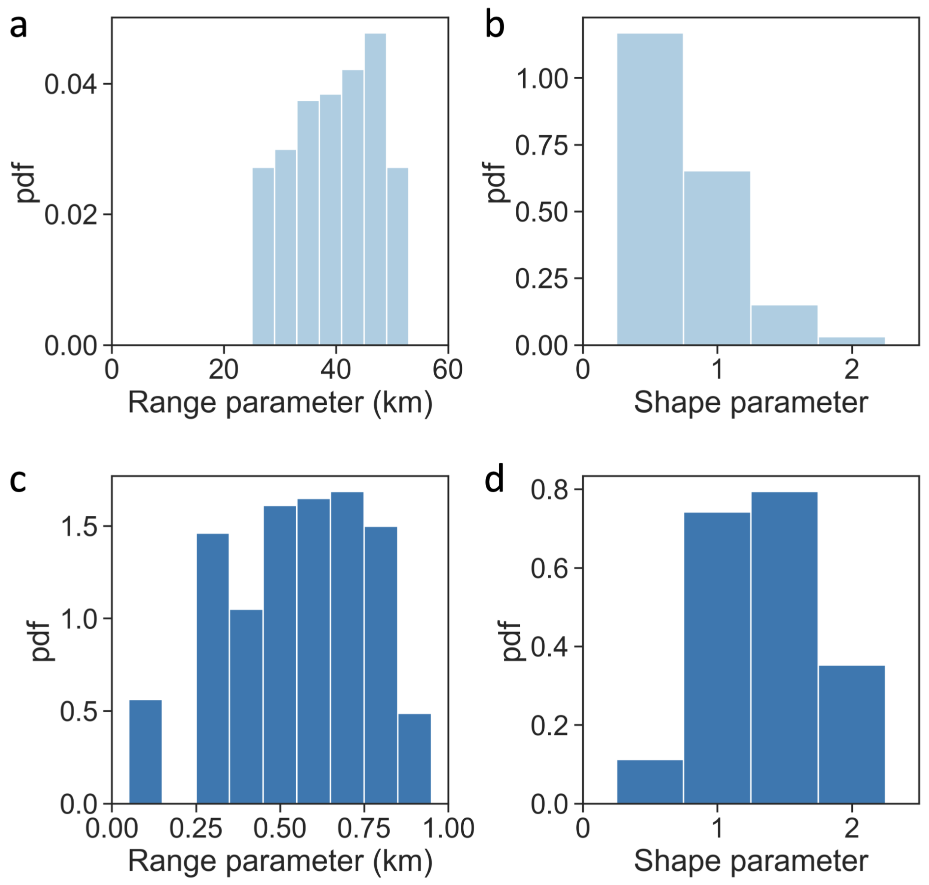

Supplementary Figure 5 | Probabilistic distributions of the seed dispersal kernel parameters across the top 5% ensembles that match with observation. Parameters of the (a, b) long-distance and (c, d) short-distance dispersal kernels.

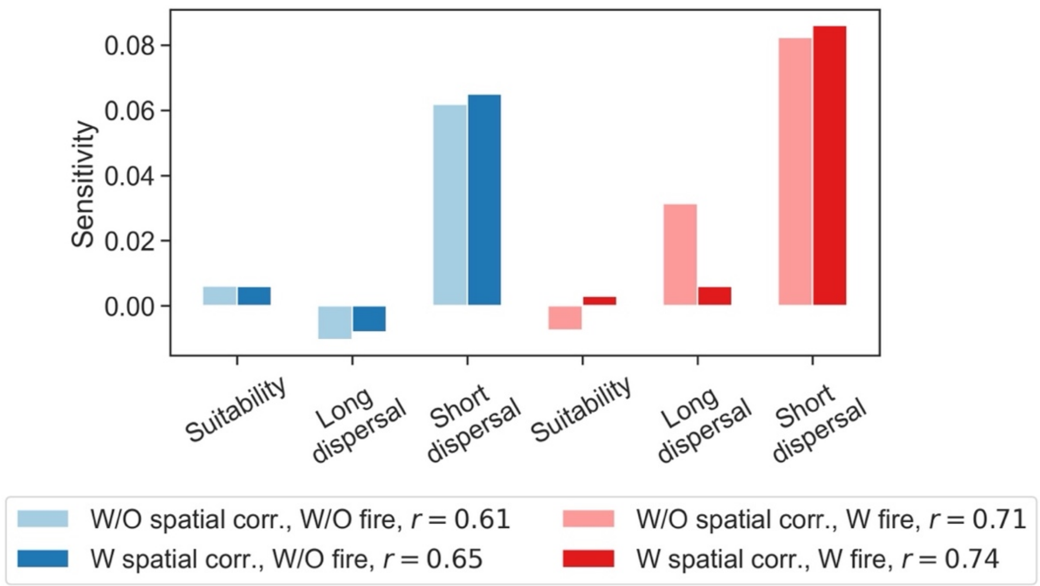

Supplementary Figure 6 | Sensitivities of shrub expansion to the environmental suitability and dispersal with and without considering spatial correlation. Considering spatial correlation (darker colors) improves the correlation between estimated and observed shrub expansion (r) from 0.61 to 0.65 for areas without fire and from 0.71 to 0.74 for areas with fire, respectively. The relative sensitivities remained similar.

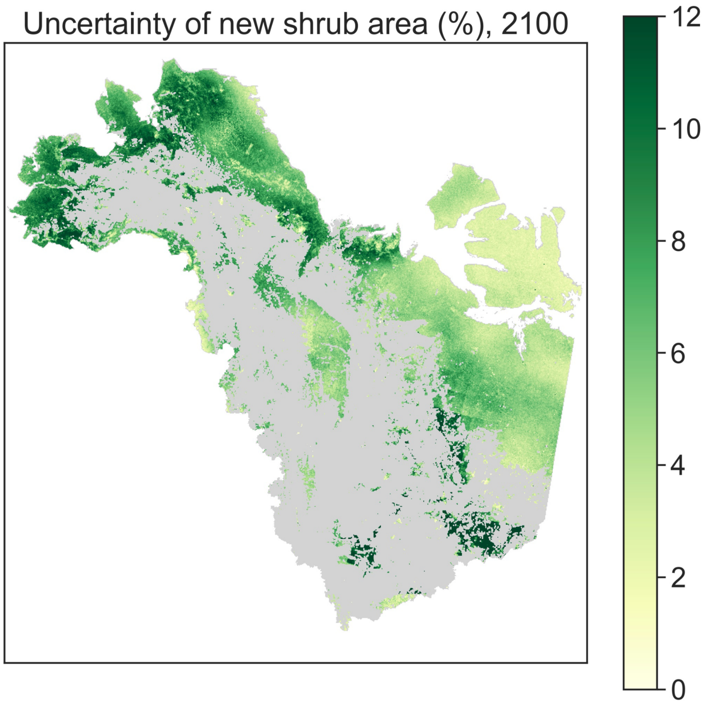

Supplementary Figure 7 | Uncertainty range of estimated new shrub area in 2100.

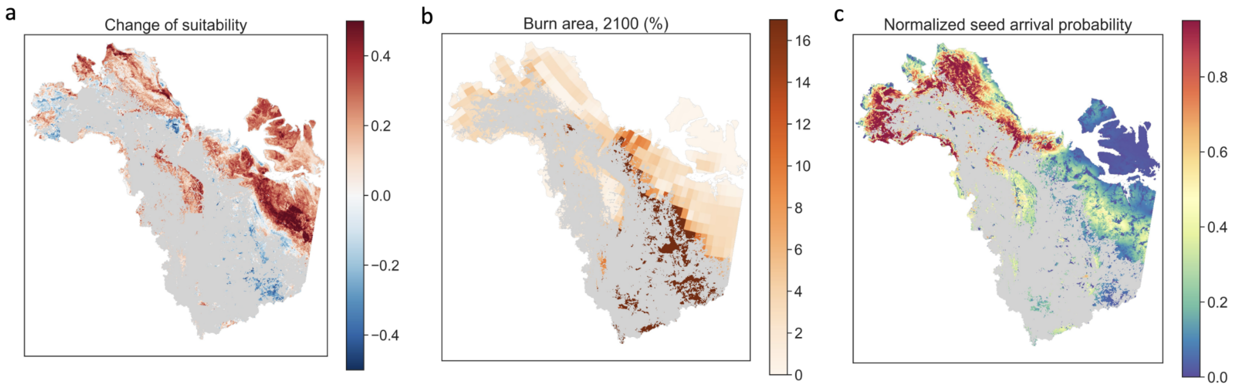

Supplementary Figure 8 | Spatial patterns of environmental suitability change from 2014 to 2100, 30-year cumulative burn area by 2100 projected in CMIP6, and seed arrival probability. Seed arrival probability includes both short and long distance dispersal and is normalized to the scale of 0-1.

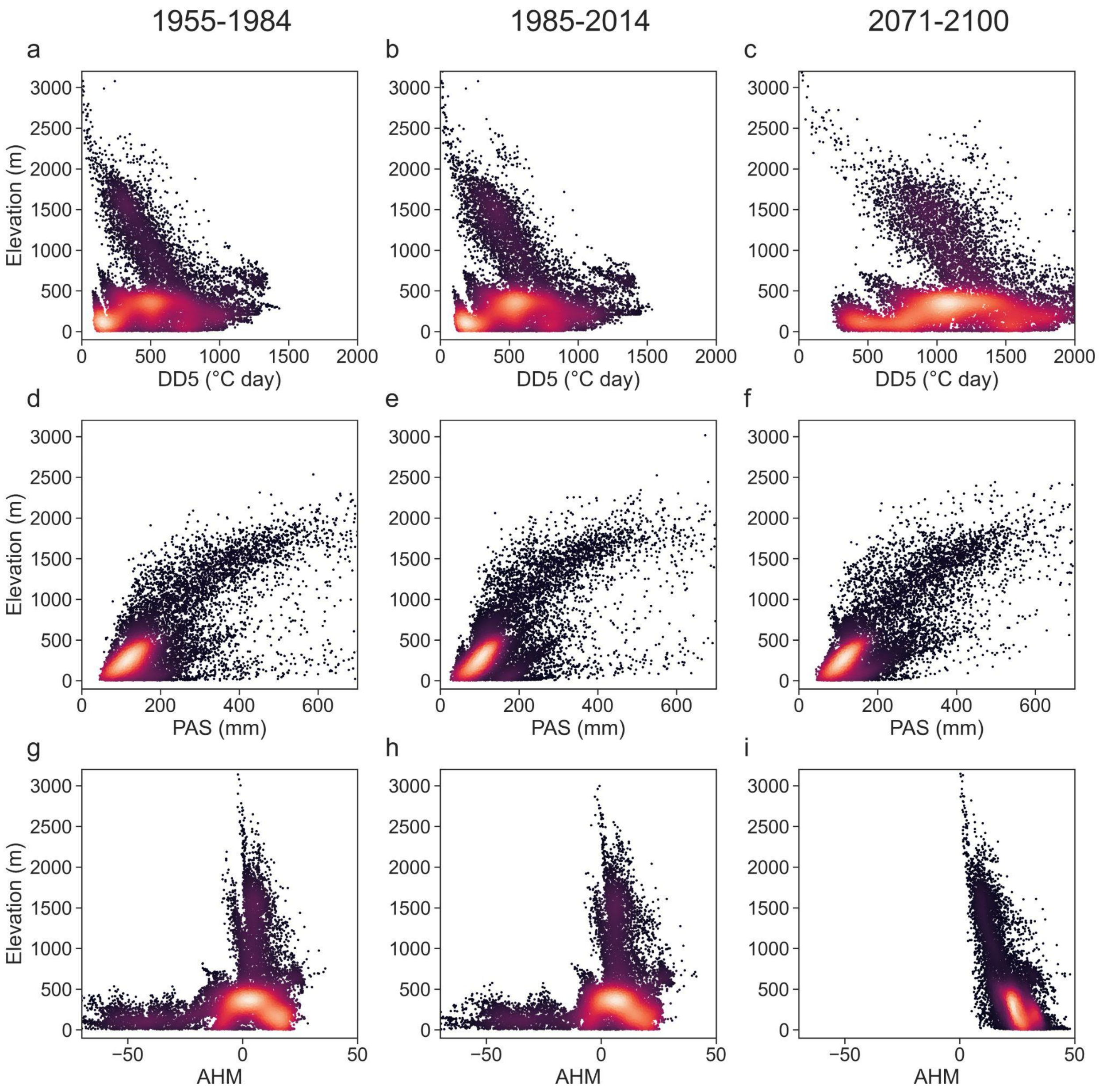

Supplementary Figure 9 | Historical and projected bioclimatic conditions and elevation across the domain. Joint distribution of (a-c) degree-days above 5°C and elevation, (d-f) annual precipitation as snow and elevation, (g-i) annual heat moisture index and elevation during (a, d, g) 1955-1984, (b, e, h) 1985-2014, and (c, f, i) 2071-2100. Brighter colors represent higher point density.

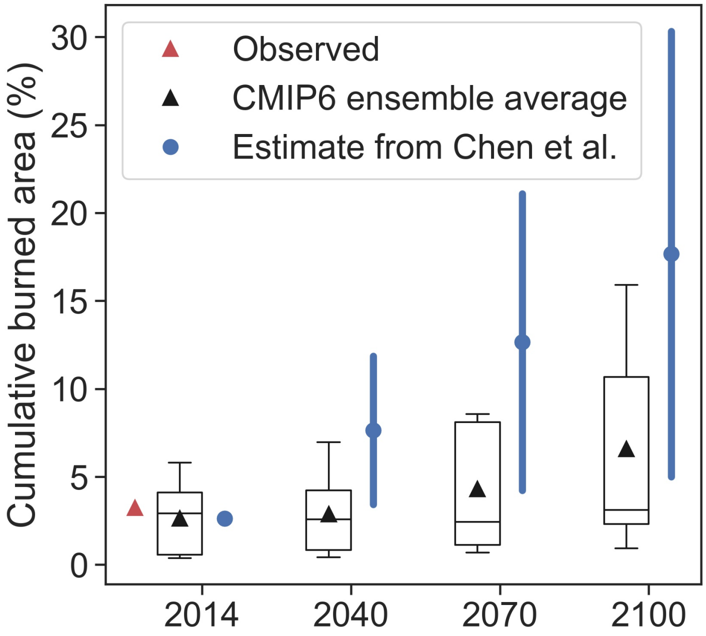

Supplementary Figure 10 | Domain average of observed and projected 30-year cumulative burn area from 2014 to 2100 based on CMIP6 and Chen et al. (2021). Red triangle shows the domain average of the observed burn area during 1984-2014. Black triangles denote the ensemble average, the upper and lower boundaries of the boxes correspond to the 25th and 75th quantiles, and the whiskers show the range of projections across CMIP6 models. Blue dots and vertical lines represent the predicted mean and uncertainty of burn area in 2100, linearly interpolated from 2014 to 2100, based on Chen et al. (2021) where vegetation-fire feedback was considered.
