# Supplementary material for: Dispersal and fire limit Arctic shrub expansion
Source: Nat Commun. 2022 Jul 4;13:3843. doi: 10.1038/s41467-022-31597-6 (PMC9253140; doi:10.1038/s41467-022-31597-6)
Supplement: Supplementary file 3 — Reporting Summary [file 41467_2022_31597_MOESM3_ESM.pdf]

nature portfolio

Corresponding author(s): Yanlan Liu

Last updated by author(s): 06/21/2022

Reporting Summary

Nature Portfolio wishes to improve the reproducibility of the work that we publish. This form provides structure for consistency and transparency in reporting. For further information on Nature Portfolio policies, see our Editorial Policies and the Editorial Policy Checklist.

Statistics

For all statistical analyses, confirm that the following items are present in the figure legend, table legend, main text, or Methods section.

n/a	Confirmed	
☐	☒	The exact sample size (n) for each experimental group/condition, given as a discrete number and unit of measurement
☒	☐	A statement on whether measurements were taken from distinct samples or whether the same sample was measured repeatedly
☒	☐	The statistical test(s) used AND whether they are one- or two-sided<br>*Only common tests should be described solely by name; describe more complex techniques in the Methods section.*
☐	☒	A description of all covariates tested
☐	☒	A description of any assumptions or corrections, such as tests of normality and adjustment for multiple comparisons
☐	☒	A full description of the statistical parameters including central tendency (e.g. means) or other basic estimates (e.g. regression coefficient) AND variation (e.g. standard deviation) or associated estimates of uncertainty (e.g. confidence intervals)
☒	☐	For null hypothesis testing, the test statistic (e.g. F, t, r) with confidence intervals, effect sizes, degrees of freedom and P value noted<br>*Give P values as exact values whenever suitable.*
☒	☐	For Bayesian analysis, information on the choice of priors and Markov chain Monte Carlo settings
☒	☐	For hierarchical and complex designs, identification of the appropriate level for tests and full reporting of outcomes
☐	☒	Estimates of effect sizes (e.g. Cohen's d, Pearson's r), indicating how they were calculated

Our web collection on statistics for biologists contains articles on many of the points above.

Software and code

Policy information about availability of computer code

Data collection	All datasets were downloaded from the public repositories specified in the methods. No software was used for data collection.
Data analysis	The terrain analysis software RichDEM in Python was used to derive additional topographic conditions from elevation. The R package biomod2 (version 3.5.1) was used to estimate environmental suitability. The image processing software of scipy.ndimage in Python was used to calculate seed arrival probability. The spatial regression was conducted using the R package spBayes (version 0.4-6). Demo code to compute suitability and seed dispersal is publicly available at https://github.com/YanlanLiu/arctic_shrub_expansion.

For manuscripts utilizing custom algorithms or software that are central to the research but not yet described in published literature, software must be made available to editors and reviewers. We strongly encourage code deposition in a community repository (e.g. GitHub). See the Nature Portfolio guidelines for submitting code & software for further information.

Data

Policy information about availability of data

All manuscripts must include a data availability statement. This statement should provide the following information, where applicable:

- Accession codes, unique identifiers, or web links for publicly available datasets
- A description of any restrictions on data availability
- For clinical datasets or third party data, please ensure that the statement adheres to our policy

The Landsat-derived product of annual dominant land cover across ABoVE core domain is available at https://daac.ornl.gov/ABOVE/guides/Annual_Landcover_ABoVE.html.
The historical and projected climate conditions and the application to downscale (ClimateNA) was downloaded from http://climatena.ca/.
The ASTER elevation data was downloaded from https://lpdaac.usgs.gov/products/astgtmv003/.

Fire occurrence data was downloaded from https://daac.ornl.gov/cgi-bin/dsviewer.pl?ds_id=1564.
Projected burn area using CMIP6 models were provided by Dr. Qing Zhu.

Field-specific reporting

Please select the one below that is the best fit for your research. If you are not sure, read the appropriate sections before making your selection.

☐ Life sciences ☐ Behavioural & social sciences ☒ Ecological, evolutionary & environmental sciences

For a reference copy of the document with all sections, see nature.com/documents/nr-reporting-summary-flat.pdf

Ecological, evolutionary & environmental sciences study design

All studies must disclose on these points even when the disclosure is negative.

Study description	We use long-term high-resolution satellite imagery across Alaska and western Canada to explore control factors of shrub expansion pattern during 1984-2014. We considered (1) environmental suitability estimated using climate and topographic conditions and a random forest model, (2) seed arrival probability calculated using convolution of seed dispersal kernels, and (3) fire occurrence from Landsat-derived burn scars. The results show shrub expansion did not follow suitability but can only be explained by considering dispersal and fire. Without considering these limitations likely lead to overestimated shrub expansion and misrepresented spatial patterns.
Research sample	The entire vegetated Arctic tundra (areas not dominated by forests) in the ABoVE core study domain was analyzed given all necessary datasets are available for this region. The area of interest includes 2.85 billion 30 m by 30 m pixels.
Sampling strategy	The suitability model was trained using randomly sampled 5% of the pixels, which were rotated for uncertainty quantification. The sample size was considered sufficient as the model accurately captures shrub distribution (r>0.9). The relationships between shrub expansion and suitability, dispersal, and fire were derived using all pixels, i.e., no sampling was involved.
Data collection	Existing datasets are downloaded from the url sources indicated above by Yanlan Liu.
Timing and spatial scale	All the annual landcover, climate, and fire occurrence data span across 1984-2014 based on data availability Projected climate and fire data cover 2014-2100. All datasets cover the entire studied domain and have a 30 m spatial resolution, except for climate data (4km) and projected fire (1-2 degree).
Data exclusions	Non-vegetated areas were excluded from the analysis.
Reproducibility	Results can be reproduced using existing datasets and the code/software used. Attempt to repeat the analysis was successful.
Randomization	No randomization is involved.
Blinding	Blinding is not relevant. Examination on alternative explanations and sensitivity analysis were performed and described in the discussion and methods section.

Did the study involve field work? ☐ Yes ☒ No

Reporting for specific materials, systems and methods

We require information from authors about some types of materials, experimental systems and methods used in many studies. Here, indicate whether each material, system or method listed is relevant to your study. If you are not sure if a list item applies to your research, read the appropriate section before selecting a response.

Materials & experimental systems

n/a	Involved in the study	
☒	☐	Antibodies
☒	☐	Eukaryotic cell lines
☒	☐	Palaeontology and archaeology
☒	☐	Animals and other organisms
☒	☐	Human research participants
☒	☐	Clinical data
☒	☐	Dual use research of concern

Methods

n/a	Involved in the study	
☒	☐	ChIP-seq
☒	☐	Flow cytometry
☒	☐	MRI-based neuroimaging
